# Viroporins vs. Other Pore-Forming Proteins: What Lessons Can We Take?

**DOI:** 10.3389/fchem.2021.626059

**Published:** 2021-02-18

**Authors:** Eva Žerovnik

**Affiliations:** Department of Biochemistry and Molecular and Structural Biology, J. Stefan Institute, Ljubljana, Slovenia

**Keywords:** SARS-CoV-2 E protein, amyloid pore, channel formation, ion conductance activity, drug target

## Abstract

Pore-forming proteins (PFPs) exist in virtually all domains of life, and by disrupting cellular membranes, depending on the pore size, they cause ion dis-balance, small substances, or even protein efflux/influx, influencing cell’s signaling routes and fate. Such pore-forming proteins exist from bacteria to viruses and also shape host defense systems, including innate immunity. There is strong evidence that amyloid toxicity is also caused by prefibrillar oligomers making “amyloid pores” into cellular membranes. For most of the PFPs, a 2-step mechanism of protein-membrane interaction takes place on the “lipid rafts,” membrane microdomains rich in gangliosides and cholesterol. In this mini-review paper, common traits of different PFPs are looked at. Possible ways for therapy of channelopathies and/or modulating immunity relevant to the new threat of SARS-CoV-2 infections could be learnt from such comparisons.

## Widely Spread Phenomenon of Pore Formation

Pore-forming proteins (PFPs) appear in virtually all organisms starting from viruses and bacteria. Bacteria use pore-forming toxins (PFTs) to disrupt plasma membrane of host cells. Even though there are several structural classes of PFTs, they all make pores after oligomerization. They can form α-helical or β-barrel transmembrane channels. Many reviews of bacterial PFTs have been written; among them is a comprehensive review by Dal Peraro and van der Goot (2016). The mechanism of pore formation by bacterial PFTs and structure of the transmembrane pores have been studied extensively. It was observed that the majority of pore-forming proteins make pores composed from transmembrane β-barrels (Heuck et al., 2001) or from clusters of α-helices (Kristan et al., 2009; Kagan and Thundimadathil, 2010). The sizes of pores are in range of a few nm up to 40 nm in diameter (Bischofberger et al., 2009). These pores allow uncontrolled permeabilization of ions and small molecules and the larger pores even of proteins. Consequences of pore formation vary and depend on the number of pores present in the plasma membrane, the mechanism of membrane binding, cell type, and so forth.

Viruses, predominantly RNA viruses, also use the so-called “viroporins” to enhance pathogenic response of the host immune system and cause extensive inflammatory response (Nieto-Torres et al., 2015). Already in 2013, the channel activity of the viroporin-E protein (E for envelope) from SARS Coronavirus was studied (Aguilella et al., 2013). The E protein of the actual SARS-CoV-2 virus, similar to the one from SARS-CoV, oligomerizes into homopentamers as obtained by Sankar et al. (2020) by molecular modeling as confirmed by NOE’s contacts from heteronuclear NMR (Sarkar and Saha, 2020). Studies of structures, dynamics, and interactions with host cells of viroporins and bacterial PFTs are important as they may help in the search for novel antibacterial and antiviral therapies.

Pore formation is not only an ancient mechanism of attack, such as used by viruses, bacteria, and lower invertebrates but is also used for signaling and defense in higher organisms (Iacovache et al., 2008; Feil et al., 2010). Innate immunity has evolved from invertebrates via fish to mammals (Buchmann, 2014). Innate effector molecules are oxygen and nitrogen species, anti-microbial peptides, lectins, fibrinogen-related peptides, leucine rich repeats, pentraxins, and complement-related proteins (Buchmann, 2014). In this context, anti-microbial peptides (AMPs), also termed host defense peptides, are used by invertebrates and vertebrates, including mammals, in order to kill microbes via membrane perforation.

Amyloid-forming proteins (AFPs), involved in neurodegenerative diseases, with highest prevalence in the aging population of Alzheimer’s and Parkinson’s disease, also form transmembrane pores/channels when in oligomeric form. *In vitro* several AFPs were shown to interact with membranes and form the so-called amyloid pores (Kagan and Thundimadathil, 2010). *In vivo* situation is a bit less clear as no-one has observed any amyloid pore directly; however, they are implied from a *C. elegans* study where the membrane repair response was observed when animals were fed by human Aβ (Julien et al., 2018). Some functional proteins also can make amyloid fibrils and pores, at least *in vitro*. Such is the case with stefin B (cystatin B) (Ceru et al., 2008; Rabzelj et al., 2008) and might underlie epileptogenesis, as suggested by Surguchov et al. (2017).

There is not much difference between AMPs and amyloid toxicity as pointed out by Jang et al. (2011), who showed that fragment of protegrin forms amyloid fibrils. That a common mechanism may apply was proposed by Last and Miranker (2013). In addition, amyloid-beta (Aβ) likely possesses anti-microbial activity, which relates AD risk to microbial infection (Moir et al., 2018). Kumar et al. (2016) show a model in which soluble Aβ oligomers first bind to microbial cell wall carbohydrates via heparin-binding domain, after which growing protofibrils inhibit pathogen adhesion to host cells. Similarly, Walsh et al. (2014) report that PrP(106–126) composition is reminiscent of cationic anti-microbial peptide dermaseptin. In agreement with expectation, oligomeric PrP(106–126) inhibited the growth of BL21 *E. coli* cultures (Walsh et al., 2014).

Additional similarity between amyloid oligomers and other PFPs is a multistep mechanism of channel formation, which includes oligomerization at the plane of the membrane (Bischofberger et al., 2009).

As there seem to be some common mechanisms on the side of lipid composition and protein oligomeric structures, I hereby suggest that one needs to study and compare what is known about the pore-forming peptides from amyloid proteins, anti-microbial peptides, bacterial PFTs, and viroporins. I drop out from consideration in the mini-review bacterial PFTs, due to their complexity and different structural classes.

## Viral Pore Formation—Viroporins

RNA of several pathogenic human viruses encodes at least one viroporin. This is the case with pathogenic human influenza A virus (IAV), human immunodeficiency virus 1 (HIV-1), hepatitis C virus (HCV), and coronaviruses (CoVs), including the one responsible for the severe acute respiratory syndrome (SARS-CoV) and the other causing Middle East respiratory syndrome (MERS-CoV) (Dal Peraro and van der Goot, 2016).

As demonstrated for SARS-CoV E protein, ion conductivity (IC) activity can overstimulate host immune response, leading to cytokine storm, also reported for the SARS-CoV-2. Of importance, when virus was devoid of E protein IC activity, it proved less lethal (Nieto-Torres et al., 2014). Viral IC activity overstimulates inflammatory response by the activation of NLRP3 inflammasome. There are some promising results in search of specific inhibitors of NLRP3 inflammasome in order to reduce inflammatory responses (Coll et al., 2015).

Thus, the envelope E protein can make homo-oligomers and generate an ion channel termed viroporin. Peptides making the transmembrane domain of E protein were synthesized, and their oligomerization was studied. It was shown that E protein can form dimers, trimers, and pentamers. When SARS-CoV E protein was expressed in Sf9 insect cells, it formed multimeric homo-oligomers. By mutations of hydrophobic residues in the TMD with charged residues, monomers were obtained. In more detail, mutations of the TMD residues asparagine 15 (N15) to alanine (N15A) and valine 25 (V25) to phenylalanine (V25F) were found to abolish the IC activity of CoV E viroporin, confirming that this activity depends on its homopentameric conformation. The ability of CoV E protein to assemble into homopentamers is clearly important for the functional CoV E viroporin (Schoeman and Fielding, 2019).

More studies have been performed recently on the structure and potential drug binding sites of the E protein from CoV-2 (Mandala et al., 2020). The orientation of five-helix bundle of the transmembrane region of the E protein in lipid bilayers was determined by solid-state NMR. It provides explanation for how Ca^2+^ ions could enter and how to block this activity, which leads to inflammasome activation (Coll et al., 2015).

For big DNA viruses and smaller RNA viruses, host cellular double membrane invaginations from ER, Golgi, and autophagosomes are used for viral transport and replication (the so-called virus factories), in a similar way as for protein aggregates removal by autophagy. There are some parallels here again with amyloid-forming proteins (see the following section). The role of cholesterol and gangliosides rich lipid rafts has been reported for both cases; it is known that disruption of the lipid rafts causes a significant reduction of viral RNA production.

*In silico* approaches to detect inhibitors of the human SARS-CoV-2 E protein ion channel activity have already led to some possible drugs (Gupta et al., 2020).

## Anti-Microbial Peptides and Amyloid Toxins: Two Sides of the Same Coin

On one hand are anti-microbial peptides (AMPs), also termed host defense peptides, used by invertebrates and vertebrates, including mammals. By perforating microbial membranes, AMPs act as potent, broad spectrum antibiotics against bacteria, fungi, and some (enveloped) viruses. Structurally they can be classified into three major groups: peptides with an α-helical conformation (e.g., insect cecropins, magainins), cyclic peptides with pairs of cysteine residues (e.g., defensins, protegrins), and peptides rich in some amino acid residues (e.g., proline rich, histidine rich). Most AMPs are proteins of <25 kDa and adopt amphipathic structures, which contribute to their interaction with anionic membranes (Bulet et al., 2004). It was shown that protegrins are able to make channels (Sokolov et al., 1999; Capone et al., 2010).

Pore-forming proteins also play an important role in innate immunity, such as the case with perforin 1 (perforating extracellular bacteria), perforin 2 (perforating bacteria which entered cells by endosomes), and membrane attack complex of the complement, with perforin-like D9 component (Voskoboinik et al., 2006; Rosado et al., 2008).

On the other hand, prefibrillar oligomers of many amyloid-forming proteins can make the so-called amyloid pores into membranes and exert cyto-toxicity. The oligomeric prefibrillar state, either, on the way to amyloid fibrils or sometimes off-pathway, after a temporary α to β secondary transition, usually adopts β-barrel transmembrane pore conformation. Amyloid pores can disrupt plasma membrane and intracellular membranes among them mitochondrial (Squier, 2001; Pagani and Eckert, 2011). For example, mitochondrial dysfunction in PD may be due to cardiolipin-promoted perforation of mitochondrial membranes by α-synuclein oligomers (Ghio et al., 2019).

A recent review by Lee et al. (2020) tries to connect properties of AMPs and AFPs, especially shorter fragments or peptides of AFPs, like Aβ and amylin. They conclude: “In fact, a large number of naturally occurring AMPs including LL37, lysozyme, protegrin-1, plant defensins, temporins, etc., form amyloid fibrils, oligomerise, and interact with membranes, causing membrane permeation by similar mechanisms to amyloid pores.”

## More on Amyloid Pores: Is There a Common Mechanism for AMPs Pores?

Morphologically and structurally amyloid pores are similar to pores formed by other pore-forming proteins (Parker and Feil, 2005; Anderluh and Lakey, 2008). They have been detected in the case of at least 12 amyloid-forming proteins, ranging from typical globular to intrinsically disordered proteins or proteolytic fragments of the amyloidogenic proteins. They are in general quite large (diameter of 3–10 nm) and rather non-selective (Butterfield and Lashuel, 2010; Kagan and Thundimadathil, 2010). Lipid components, such as sphingolipin and cholesterol, part of the lipid rafts, facilitate the conformational change of the amyloid pores from natively unfolded into α-helix and/or β-sheet-rich structures (Butterfield and Lashuel, 2010).

Amyloid pores have been observed by oligomers of α-synuclein, Aβ, and prion, among others. Even though the oligomers have not been visualized in interaction with cellular membranes *in vivo*, they are indirectly indicated by pore-like activities such as Ca^2+^ entry, mitochondrial ROS increase, and nuclear pore damage. However, recently the channel activity of Aβ was observed in extracted cells membranes (Bode et al., 2017). Moreover, what is even more convincing, an animal model of *C. elegans* showed membrane defense response in this worm when challenged by human Aβ (fed by *E. coli* bacteria expressing the Aβ peptide) (Julien et al., 2018). Current understanding about the relative toxicity of endogenous soluble α-synuclein oligomers and multimers and their cross-reactivity with Tau and Aβ in different neurodegenerative diseases is reviewed by Kayed et al. (2020).

To determine the structure of oligomers making amyloid pores has also been challenging; however, atomic force microscopy (AFM) has provided some insight on the pore structure (Lin et al., 2001). Amyloid-beta (Aβ), 40 or 42 long peptide-forming plaques in Alzheimer’s disease, has been extensively studied. Aβ (1-42) in a planar lipid bilayer revealed multimeric (tetrameric, pentameric, and hexameric) channel-like structures. In accordance, electrophysiological recordings demonstrated the presence of multiple single channel currents. At the cellular level, Aβ (1-42) incorporation increased calcium influx and induced aberrant neuritic growth (Lin et al., 2001). A very recent paper by Ciudad et al. (2020) described a molecular dynamics study of insertion of Aβ (1-42) tetramers and octamers in lipid bilayers. A mechanism of membrane disruption in which water permeation occurred through lipid-stabilized pores has been revealed.

Di Scala et al. (2016) proposed a common molecular mechanism of amyloid pore formation by Aβ and alpha-synuclein (αS). They have compared a panel of amyloid-forming fragments of the above-mentioned proteins and arrived at conclusion that 2-step mechanism applies, whereas each of the gangliosides and cholesterol components of lipid membranes interacts with specific structural motifs of Aβ and αS. Whether this is a universal mechanism applying to other amyloid toxins remains to be seen.

Fusco et al. (2016) characterized membrane bound αS. Despite the biological relevance, the structural details of the membrane-bound oligomer of αS remain elusive. It is difficult to isolate a well-defined and stable oligomer and also difficult to study it in cells. The authors used solid state NMR and restrained MD simulations to refine the structure of the N-terminal (1-30 a. acids) of αS bound to synaptic-like membranes. The results indicate that the first 12 residues of αS are key to anchoring the protein to lipid surface. In order to improve the study bearing in mind that αS pore could be in the soluble fraction, Fusco et al. (2017) used solid state and solution NMR to determine structural constraints of αS membrane interaction. The structured region strongly inserted into lipid bilayers and disrupted their integrity, leading finally to cell death. Mutations which prevented membrane interaction also prevented toxicity. The authors reported two types of oligomers; the ones with more β-structure and deeper membrane insertion/disruption proved toxic in distinction to surface bound oligomers.

Canale with coworkers studied non-pathological bacterial protein HypF-N as a model for amyloid induced toxicity (Oropesa-Nuñez et al., 2016; Oropesa-Nuñez et al., 2018). They differentiated between toxic and non-toxic HypF-N oligomers and used AFM to observe their interaction with lipid bilayers. Their findings support the notion that GM1 ganglioside mediates the oligomer-membrane interaction.

Scheme was taken from Žganec and Žerovnik (2014).

## Discussion

This mini-review aims to compare features and mechanisms of pore formation by amyloid-forming proteins (AFPs), that is, their membrane perforating oligomers, anti-microbial peptides (AMPs), also called defense peptides of the innate immunity system and viroporins, and transmembrane short viral envelope proteins (E protein), helping spread certain viruses, among them the coronavirus SARS-CoV-2.

One should be able to derive common structural traits and interaction mechanism of some AFPs, AMPs, and viroporins, which would include oligomerization, alignment of α-helices against lipid surface (on acidic phospholipids, initially driven by electrostatics), and transition of the “pre-pore” into β structure and making a pore (Omersa et al., 2019). By finding common mechanisms, perhaps one could design common means of defense and augment anti-viral and anti-amyloid therapies. By stabilizing membranes, inhibiting the process of pore formation by small drugs/peptides competing with ganglioside and cholesterol binding sites or inhibiting channel conductance might be a possible therapeutic way to attack such broad spectrum of disease (Scott and Griffin, 2015). Compounds blocking channel activity by Aβ oligomers have been reported for a mouse model of AD (Martinez Hernandez et al., 2018).

Out of curiosity, perhaps, we have previously compared Aβ, part of prion protein and part of our model amyloid-forming protein, stefin B (cystatin B) (Yoichi et al., 2005), which also (when in prefibrillar oligomeric form) makes pores into acidic phospholipid membranes as our *in vitro* studies show (Ceru and Zerovnik, 2008; Rabzelj et al., 2008). In the Clustal alignment, the α-helix and first β-strand of stefin B showed low similarity with parts of prion and amyloid-beta, including the protease binding site QVVAG. This comparison might seem superficial. However, I suggest to compare the sequences of more pore-forming peptides and to use more sophisticated methods of prediction and sequence comparative analysis, for example, those used in Venko et al., submitted to Frontiers in Mol. Neuroscience.

**FIGURE 1:**
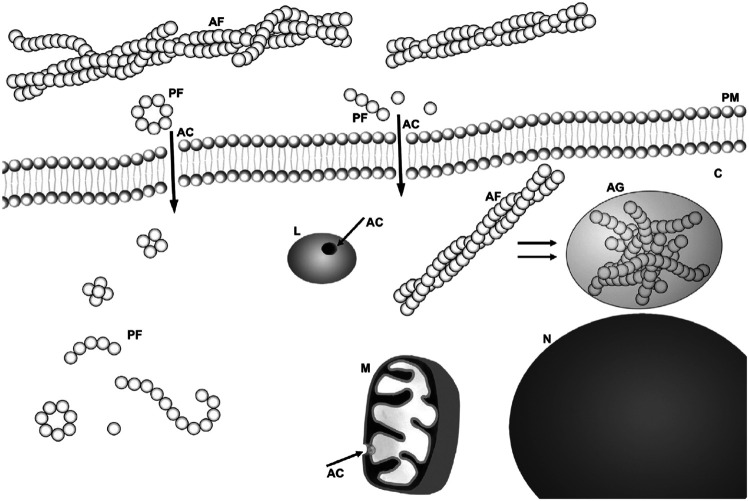
Scheme of possible sites for amyloid “toxins”: membrane interaction/perforation where AF stands for amyloid fibrils, PF for annular and other kind of protofibrils, AC for amyloid channel, M for mitochondria, N for nucleus, and L for lysosome. Taken from Žganec and Žerovnik (2014), copyright to Elsevier.
